# Crystal structure, DFT and MEP study of (*E*)-2-{[(3-chloro­phen­yl)imino]­meth­yl}-6-methyl­phenol

**DOI:** 10.1107/S2056989019017353

**Published:** 2020-01-07

**Authors:** Hanife Saraçoğlu, Onur Erman Doğan, Tuğgan Ağar, Necmi Dege, Turganbay S. Iskenderov

**Affiliations:** a Ondokuz Mayıs University, Educational Faculty, Department of Mathematic and Science Education, 55139, Samsun, Turkey; b Ondokuz Mayıs University, Faculty of Arts and Sciences, Department of Chemistry, 55139, Samsun, Turkey; c Yeditepe University, Department of Chemical Engineering, 34755, Istanbul, Turkey; d Ondokuz Mayıs University, Faculty of Arts and Sciences, Department of Physics, 55139, Samsun, Turkey; e Department of Chemistry, Taras Shecchenko National University of Kyiv, 64, Vladimirska Str., Kiev 01601, Ukraine

**Keywords:** energy gap, Schiff base, anti-bacterial agent, frontier mol­ecular orbitals, crystal structure

## Abstract

In the crystal structure of the title compound, C_14_H_12_ClNO, the mol­ecules are linked through C—H⋯O hydrogen bonds and C—H⋯π inter­actions, forming chains parallel to the [010] direction. π–π inter­actions and intra­molecular hydrogen bonds are also observed. The mol­ecular geometry of the title compound in the ground state has been calculated using density functional theory at the B3LYP level with the 6–311++G(2*d*,2*p*) basis set. Additionally, frontier mol­ecular orbital and mol­ecular electrostatic potential map analyses were performed.

## Abstract 2

In the crystal structure of the title compound, mol­ecules are linked through C—H⋯O hydrogen bonds and C—H⋯π inter­actions, forming chains parallel to the [010] direction. The mol­ecular geometry in the ground state was been calculated using DFT. Additionally, frontier mol­ecular orbital and mol­ecular electrostatic potential map analyses were performed.

## Chemical context   

Schiff bases, known as anils, imines or azomethines, have recently received considerable attention because of their good performance in coordination chemistry and anti-bacterial, anti-cancer and herbicidal applications (Piotr *et al.*, 2009 ▸; Schiff, 1864 ▸). The presence of a lone pair of electrons in an *sp* ^2^-hybridized orbital on the nitro­gen atom of the azomethine group is of considerable chemical and biological importance (Sinha *et al.*, 2008 ▸). In a continuation of our inter­est in the chemical, herbicidal and biological properties of Schiff bases we synthesized the title compound, (I), as a potential anti-bacterial agent (Yılmaz *et al.*, 2012 ▸).
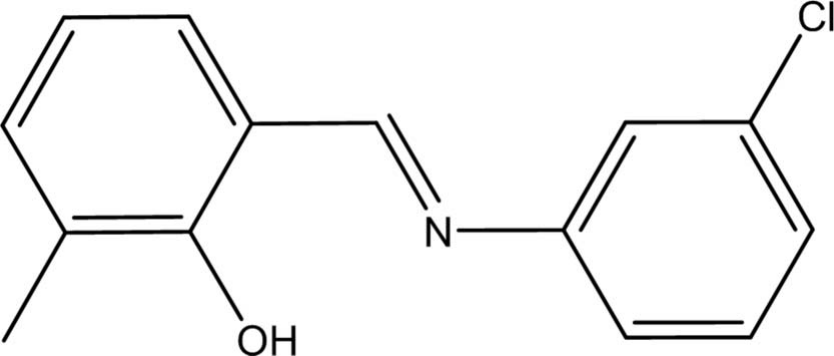


We report herein the synthesis, crystal structure and quantum chemical computational studies of the Schiff base compound, (I).

## Structural commentary   

**Figure 1:**
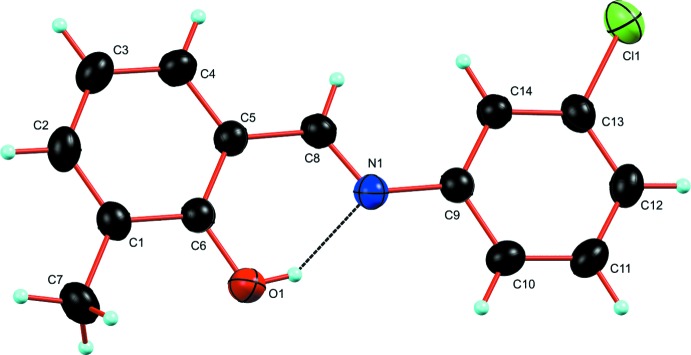
A view of the mol­ecular structure of (I) with the atom labelling. The dotted line indicates the intra­molecular O—H⋯N hydrogen bond. Displacement ellipsoids are shown at the 40% probability level.

The structure of the title compound (I) is shown in Fig. 1 ▸. It crystallizes in the ortho­rhom­bic space group *Pbca* with eight mol­ecules in the unit cell. The mol­ecular structure has two planar rings. The whole mol­ecule is approximately planar, with a maximum deviation of −0.0236 (12) Å from planarity for the C8 atom of Schiff base. The title compound displays an *E* configuration with respect to the C8=N double bond. The dihedral angle between the two phenyl ring planes is 0.34 (9)° and the C5—C8—N1—C9 torsion angle is −179.81 (15)°. The planar mol­ecular conformation is stabilized by the intra­molecular O1—H1⋯N1 hydrogen bond, which forms an *S*(6) motif.

## Supra­molecular features   

**Figure 2:**
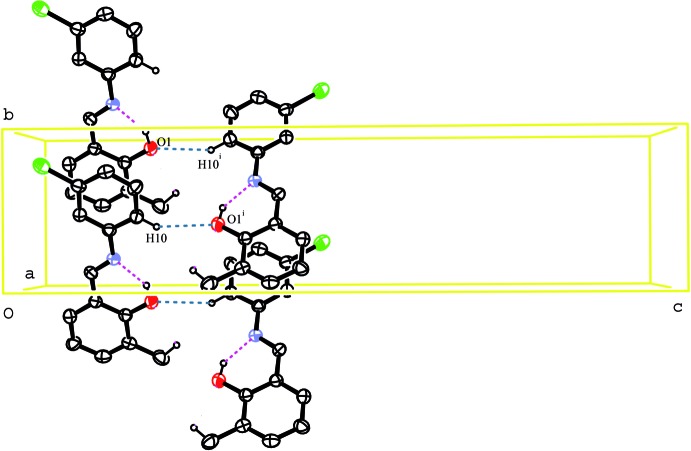
Diagram showing the hydrogen-bonding inter­actions in (I). Displacement ellipsoids are drawn at the 40% probability level. Symmetry code: (i) −*x* + 1, *y* + 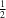, −*z* + 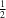.

**Figure 3:**
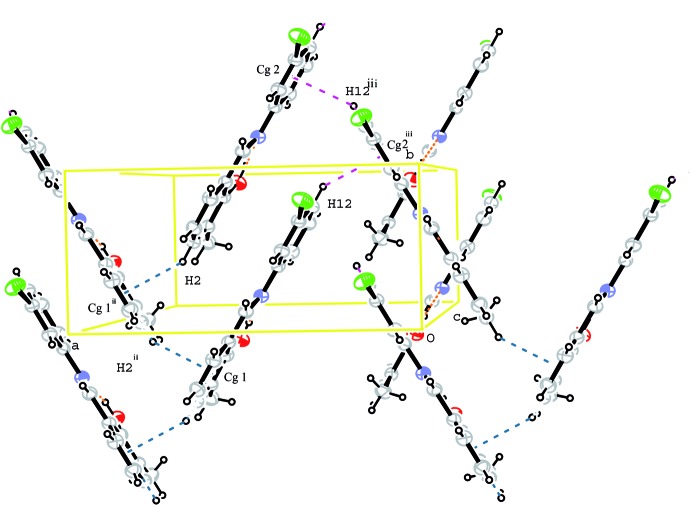
A partial packing diagram for (I) showing the C—H⋯π inter­actions as dashed lines. Symmetry codes: (ii) −*x* + 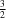, *y* − 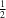, *z*; (iii) −*x* + 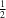, *y* + 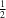, *z*.. *Cg*1 and *Cg*2 are the centroids of the methyl­phenol and chloro­phenyl rings, respectively.

In the crystal, the mol­ecules are linked by C10—H10⋯O1 hydrogen bonds (Table 1 ▸), generating a 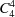
(16) chain running parallel to the [010] direction (Fig. 2 ▸). C—H⋯π inter­actions occur between the two phenyl rings (Fig. 3 ▸, Table 1 ▸). π–π stacking inter­actions [centroid–centroid distance = 3.6389 (11) Å] between the chloro­phenyl and methyl­phenol rings are also observed.

## Database survey   

A search of the Cambridge Structural Database (CSD, version 5.40; update Nov. 2018; Groom *et al.*, 2016 ▸) gave eighteen hits for the (*E*)-2-{[(3-chloro­phen­yl)imino]­meth­yl}-6-methyl­phen­ol structure. With a value of 1.271 (2) Å, the N1—C8 bond in the title compound (I) is the same length within standard uncertainties as those in the structures of 2-[(*E*)-(5-chloro-2-methyl­phen­yl)imino­meth­yl]-4-methyl­phenol (AFILAE; Zheng, 2013*b* ▸), 2,4-di­bromo-6-{[(5-chloro-2-methyl­phen­yl)imino]­meth­yl}phen­ol (AGEGUQ; Zheng, 2013*a* ▸), 2-[(*E*)-(2,4,6-tri­chloro­phen­yl)imino­meth­yl]phenol (AWUSIV; Fun *et al.*, 2011 ▸), *N*-(2-methyl-5-chloro­phen­yl)salicylaldimine (BEYQEB; Elmalı & Elerman, 1998 ▸), (*E*)-2-[(3-chloro­phenyl­imino)­meth­yl]-4-meth­oxy­phenol (DUBNAQ; Özek *et al.*, 2009 ▸), 3-{(*E*)-[(3,4-di­chloro­phen­yl)imino]­meth­yl}benzene-1,2-diol (MOYHAL; Tahir *et al.*, 2015 ▸) and *N*-(3-chloro­phen­yl)salicylaldimine (NADZUO; Karakaş *et al.*, 2004 ▸) where the C=N bond length varies from 1.266 (4) to 1.290 (3) Å. These structures also have an intra­molecular O1—H1⋯N1 hydrogen bond resulting in the formation of a six-membered ring and exhibit an *E* configuration.

## Frontier mol­ecular orbital analysis   

**Figure 4:**
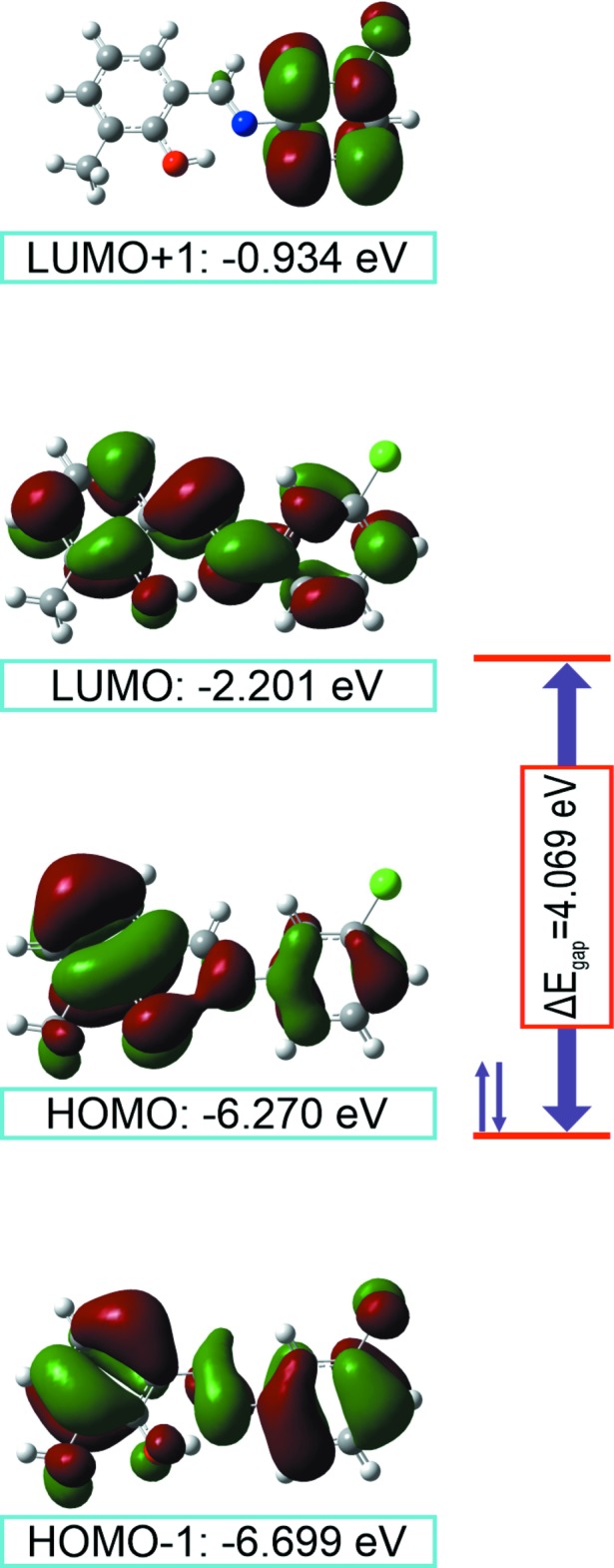
Plots of the frontier orbitals and the energy gap for (I).

The frontier mol­ecular orbitals are important in the deter­min­ation of the optical, electronic and anti-corrosion properties of a mol­ecular system (Koepnick *et al.*, 2010 ▸; Solomon *et al.*, 2012 ▸; Jafari *et al.*, 2013 ▸). A mol­ecule with a small frontier orbital gap is more polarizable than one with a large gap and is considered a soft mol­ecule because of its high chemical reactivity and low kinetic stability (Prabavathi *et al.*, 2015 ▸). The energy levels of the HOMO (highest occupied mol­ecular orbital), HOMO-1, LUMO (lowest occupied mol­ecular orbital) and LUMO+1 orbitals calculated at the B3LYP/6-311++G(2*d*,2*p*) level (Frisch *et al.*, 2009 ▸; Dennington *et al.*, 2007 ▸) for (I) are shown in Fig. 4 ▸. The HOMO, HOMO-1 and LUMO orbitals are delocalized over the two phenyl rings connected by a Schiff base bridge and HOMO and HOMO-1 can be said to be π-bonding orbitals. The LUMO+1 orbitals are delocalized on the chloro­phenyl ring and the C atom of the Schiff base. LUMO and LUMO+1 orbitals exhibit π* anti­bonding character. The energy gap of (I) is 4.069 eV. The other mol­ecular orbital energies are shown in Fig. 4 ▸. Electron affinity (*A*) and ionization potential (*IP*) can be defined as *A* = −*E*_LUMO_ and *IP* = −*E*_HOMO_. Additionally, these values can also be used calculate the electronegativity (χ), chemical hardness (η) and chemical softness (S) (Prabavathi *et al.*, 2015 ▸; Karunakaran & Balachandran, 2014 ▸). For the title compound (I), *A* = 2.201 eV, *IP* = 6.270 eV, χ = 4.236 eV, η = 2.035 eV, and *S* = 0.246 eV.

## Mol­ecular electrostatic potential surface analysis   

**Figure 5:**
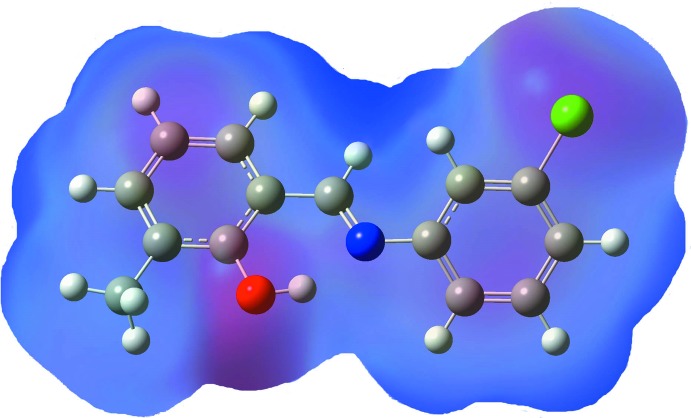
Mol­ecular electrostatic potential (MEP) map calculated at the B3LYP/6–311++G(2*d*,2*p*) level.

The analysis of a three-dimensional plot of the mol­ecular electrostatic potential (MEP) surface is a technique for mapping the electrostatic potential onto the isoelectronic density surface, providing information about the reactive sites. The surface simultaneously displays mol­ecular size and shape and the electrostatic potential value. In the colour scheme adopted, red indicates an electron-rich region with a partial negative charge and blue an electron-deficient region with partial positive charge, light blue indicates a slightly electron-deficient region, yellow a slightly electron-rich region and green a neutral region (Politzer *et al.*, 2002 ▸). The MEP map of (I) was obtained by the B3LYP/6-311++G(2*d*,2*p*) method. In Fig. 5 ▸, it is shown that (I) has two possible sites of electrophilic attack. The negative region is localized on the protonated oxygen atom of methyl­phenol ring, O1, with a minimum value of −0.031 a.u. Positive potential sites of the compound are around hydrogen atoms. However, the maximum positive region is localized on the hydrogen atom bonded to the C atom forming the Schiff base, which can be considered as one possible site for nucleophilic attack, with a maximum value of 0.027 a.u.

## Synthesis and crystallization   

A mixture of 2-hy­droxy-3-methyl­benzaldehyde (34.0 mg, 0.25 mmol) and 4-chloro­aniline (31.9 mg, 0.25 mmol) was stirred with ethanol (30 mL) at 377 K for 4 h, affording the title compound (43.0 mg, yield 70%, m.p. 362–364 K). Single crystals suitable for X-ray measurements were obtained by recrystallization from methanol at room temperature.

## Refinement   

Crystal data, data collection and structure refinement details are summarized in Table 2 ▸. H atoms were placed in calculated positions and refined using a riding model with C—H = 0.93–0.96 Å, *U*_iso_(H) = 1.2–1.5*U*_eq_(C).

## Supplementary Material

Crystal structure: contains datablock(s) I. DOI: 10.1107/S2056989019017353/mw2154sup1.cif

Structure factors: contains datablock(s) I. DOI: 10.1107/S2056989019017353/mw2154Isup2.hkl

Supporting information file. DOI: 10.1107/S2056989019017353/mw2154Isup3.cml

CCDC reference: 1974885

Additional supporting information:  crystallographic information; 3D view; checkCIF report

## Figures and Tables

**Table 1:** Hydrogen-bond geometry (Å, °) *Cg*1 and *Cg* are the centroids of the C1–C6 and C9–C14 rings, respectively.

*D*—H⋯*A*	*D*—H	H⋯*A*	*D*⋯*A*	*D*—H⋯*A*
O1—H1⋯N1	0.82	1.88	2.605 (2)	148
C10—H10⋯O1^i^	0.93	2.56	3.402 (2)	151
C2—H2⋯*Cg*1^ii^	0.93	2.75	3.561 (2)	147
C12—H12⋯*Cg*2^iii^	0.93	2.78	3.589 (2)	147

Symmetry codes: (i) 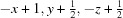; (ii) 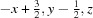; (iii) 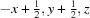.

**Table 2:** Experimental details

Crystal data	
Chemical formula	C_14_H_12_ClNO
*M*_r_	245.70
Crystal system, space group	Orthorhombic, *P* *b* *c* *a*
Temperature (K)	296
*a*, *b*, *c* (Å)	14.0717 (8), 6.4811 (4), 26.767 (2)
*V* (Å^3^)	2441.1 (3)
*Z*	8
Radiation type	Mo *K*α
μ (mm^−1^)	0.29
Crystal size (mm)	0.45 × 0.43 × 0.38

Data collection	
Diffractometer	Stoe *IPDS* 2
Absorption correction	Integration (*X-RED32*; Stoe & Cie, 2002 ▸)
*T*_min_, *T*_max_	0.820, 0.907
No. of measured, independent and observed [*I* > 2σ(*I*)] reflections	10011, 2052, 1669
*R*_int_	0.033
(sin θ/λ)_max_ (Å^−1^)	0.586

Refinement	
*R*[*F* ^2^ > 2σ(*F* ^2^)], *wR*(*F* ^2^), *S*	0.039, 0.117, 1.05
No. of reflections	2052
No. of parameters	155
H-atom treatment	H-atom parameters constrained
Δρ_max_, Δρ_min_ (e Å^−3^)	0.16, −0.24

Computer programs: *X-AREA* and *X-RED32* (Stoe & Cie, 2002 ▸), *SHELXT2018* (Sheldrick, 2015*a* ▸), *SHELXL2018* (Sheldrick, 2015*b* ▸), *ORTEP-3 for Windows* and *WinGX* (Farrugia, 2012 ▸), *Mercury* (Macrae *et al.*, 2006 ▸) and *PLATON* (Spek, 2009 ▸).

## supplementary crystallographic information

### Crystal data

**Table:**

C_14_H_12_ClNO	*D*_x_ = 1.337 Mg m^−^^3^
*M**_r_* = 245.70	Mo *K*α radiation, λ = 0.71073 Å
Orthorhombic, *P**b**c**a*	Cell parameters from 15471 reflections
*a* = 14.0717 (8) Å	θ = 1.5–25.2°
*b* = 6.4811 (4) Å	µ = 0.29 mm^−^^1^
*c* = 26.767 (2) Å	*T* = 296 K
*V* = 2441.1 (3) Å^3^	Prism, orange
*Z* = 8	0.45 × 0.43 × 0.38 mm
*F*(000) = 1024	

### Data collection

**Table:**

Stoe IPDS 2 diffractometer	2052 independent reflections
Radiation source: sealed X-ray tube, 12 x 0.4 mm long-fine focus	1669 reflections with *I* > 2σ(*I*)
Plane graphite monochromator	*R*_int_ = 0.033
Detector resolution: 6.67 pixels mm^-1^	θ_max_ = 24.6°, θ_min_ = 1.5°
rotation method scans	*h* = −16→16
Absorption correction: integration (X-RED32; Stoe & Cie, 2002)	*k* = −6→7
*T*_min_ = 0.820, *T*_max_ = 0.907	*l* = −30→31
10011 measured reflections	

### Refinement

**Table:**

Refinement on *F*^2^	Primary atom site location: other
Least-squares matrix: full	Hydrogen site location: inferred from neighbouring sites
*R*[*F*^2^ > 2σ(*F*^2^)] = 0.039	H-atom parameters constrained
*wR*(*F*^2^) = 0.117	*w* = 1/[σ^2^(*F*_o_^2^) + (0.0666*P*)^2^ + 0.3364*P*] where *P* = (*F*_o_^2^ + 2*F*_c_^2^)/3
*S* = 1.05	(Δ/σ)_max_ < 0.001
2052 reflections	Δρ_max_ = 0.16 e Å^−^^3^
155 parameters	Δρ_min_ = −0.24 e Å^−^^3^
0 restraints	

### Special details

**Table:**

Geometry. All esds (except the esd in the dihedral angle between two l.s. planes) are estimated using the full covariance matrix. The cell esds are taken into account individually in the estimation of esds in distances, angles and torsion angles; correlations between esds in cell parameters are only used when they are defined by crystal symmetry. An approximate (isotropic) treatment of cell esds is used for estimating esds involving l.s. planes.

### Fractional atomic coordinates and isotropic or equivalent isotropic displacement parameters (Å^2^)

**Table:**

	*x*	*y*	*z*	*U*_iso_*/*U*_eq_	
Cl1	0.33517 (5)	0.79127 (10)	0.03649 (2)	0.0888 (3)	
C12	0.32979 (13)	0.7525 (3)	0.13616 (8)	0.0632 (5)	
H12	0.296569	0.876559	0.136229	0.076*	
C13	0.35979 (13)	0.6646 (3)	0.09213 (7)	0.0565 (5)	
C11	0.35033 (14)	0.6518 (3)	0.18009 (8)	0.0655 (5)	
H11	0.330163	0.707801	0.210266	0.079*	
C14	0.40948 (13)	0.4816 (3)	0.09088 (7)	0.0554 (5)	
H14	0.428370	0.425004	0.060536	0.067*	
C9	0.43105 (12)	0.3826 (3)	0.13554 (6)	0.0501 (4)	
C10	0.40037 (13)	0.4690 (3)	0.18002 (7)	0.0581 (5)	
H10	0.413635	0.403110	0.210087	0.070*	
C8	0.51952 (12)	0.1055 (3)	0.10169 (7)	0.0533 (4)	
H8	0.511386	0.163728	0.070226	0.064*	
N1	0.48348 (10)	0.1964 (2)	0.13929 (5)	0.0524 (4)	
C4	0.60571 (14)	−0.1837 (3)	0.06298 (7)	0.0617 (5)	
H4	0.594284	−0.124767	0.031869	0.074*	
C5	0.57270 (12)	−0.0849 (3)	0.10589 (6)	0.0507 (4)	
C3	0.65470 (14)	−0.3660 (3)	0.06587 (8)	0.0658 (5)	
H3	0.676683	−0.430204	0.037024	0.079*	
C6	0.59085 (12)	−0.1759 (3)	0.15252 (6)	0.0511 (4)	
O1	0.56117 (10)	−0.0861 (2)	0.19521 (4)	0.0668 (4)	
H1	0.533250	0.021525	0.188623	0.100*	
C2	0.67106 (13)	−0.4535 (3)	0.11231 (8)	0.0631 (5)	
H2	0.703893	−0.577825	0.114078	0.076*	
C1	0.64031 (13)	−0.3625 (3)	0.15602 (7)	0.0563 (5)	
C7	0.65775 (16)	−0.4616 (4)	0.20614 (9)	0.0763 (6)	
H7A	0.694377	−0.585261	0.201724	0.114*	
H7B	0.598004	−0.495240	0.221426	0.114*	
H7C	0.691973	−0.367515	0.227197	0.114*	

### Atomic displacement parameters (Å^2^)

**Table:**

	*U*^11^	*U*^22^	*U*^33^	*U*^12^	*U*^13^	*U*^23^
Cl1	0.1145 (5)	0.0730 (4)	0.0790 (4)	0.0241 (3)	−0.0112 (3)	0.0176 (3)
C12	0.0587 (11)	0.0503 (11)	0.0807 (15)	0.0027 (9)	0.0037 (9)	−0.0065 (10)
C13	0.0583 (10)	0.0475 (11)	0.0637 (11)	0.0008 (8)	−0.0049 (8)	0.0044 (9)
C11	0.0684 (12)	0.0645 (13)	0.0635 (12)	0.0014 (10)	0.0096 (9)	−0.0150 (10)
C14	0.0651 (11)	0.0511 (11)	0.0502 (10)	0.0032 (9)	−0.0001 (8)	0.0002 (8)
C9	0.0527 (9)	0.0450 (10)	0.0526 (9)	−0.0037 (8)	−0.0025 (7)	0.0014 (8)
C10	0.0644 (11)	0.0594 (12)	0.0506 (10)	−0.0040 (9)	0.0028 (8)	−0.0030 (9)
C8	0.0620 (11)	0.0483 (10)	0.0498 (9)	−0.0007 (8)	−0.0023 (8)	0.0062 (8)
N1	0.0595 (9)	0.0461 (9)	0.0517 (8)	0.0007 (7)	−0.0021 (6)	0.0006 (7)
C4	0.0693 (11)	0.0610 (13)	0.0546 (11)	0.0007 (10)	0.0075 (9)	0.0022 (9)
C5	0.0545 (9)	0.0454 (10)	0.0520 (10)	−0.0020 (8)	0.0018 (7)	0.0011 (8)
C3	0.0676 (12)	0.0597 (12)	0.0702 (13)	0.0030 (10)	0.0129 (9)	−0.0078 (10)
C6	0.0532 (9)	0.0483 (11)	0.0518 (10)	−0.0016 (8)	−0.0018 (7)	0.0006 (8)
O1	0.0889 (10)	0.0615 (9)	0.0500 (7)	0.0148 (7)	−0.0033 (6)	0.0003 (6)
C2	0.0545 (10)	0.0492 (11)	0.0854 (14)	0.0022 (8)	0.0064 (9)	0.0017 (10)
C1	0.0521 (9)	0.0512 (11)	0.0655 (11)	−0.0010 (8)	−0.0028 (8)	0.0064 (9)
C7	0.0836 (15)	0.0674 (14)	0.0779 (14)	0.0115 (11)	−0.0107 (11)	0.0173 (11)

### Geometric parameters (Å, º)

**Table:**

Cl1—C13	1.7357 (19)	C4—C3	1.370 (3)
C12—C13	1.375 (3)	C4—C5	1.395 (2)
C12—C11	1.376 (3)	C4—H4	0.9300
C12—H12	0.9300	C5—C6	1.404 (2)
C13—C14	1.377 (3)	C3—C2	1.386 (3)
C11—C10	1.378 (3)	C3—H3	0.9300
C11—H11	0.9300	C6—O1	1.348 (2)
C14—C9	1.390 (2)	C6—C1	1.399 (3)
C14—H14	0.9300	O1—H1	0.8200
C9—C10	1.385 (2)	C2—C1	1.380 (3)
C9—N1	1.418 (2)	C2—H2	0.9300
C10—H10	0.9300	C1—C7	1.507 (3)
C8—N1	1.271 (2)	C7—H7A	0.9600
C8—C5	1.448 (2)	C7—H7B	0.9600
C8—H8	0.9300	C7—H7C	0.9600
			
C13—C12—C11	118.11 (18)	C5—C4—H4	119.4
C13—C12—H12	120.9	C4—C5—C6	118.61 (16)
C11—C12—H12	120.9	C4—C5—C8	119.96 (16)
C12—C13—C14	122.25 (18)	C6—C5—C8	121.42 (16)
C12—C13—Cl1	118.56 (15)	C4—C3—C2	119.16 (19)
C14—C13—Cl1	119.19 (15)	C4—C3—H3	120.4
C12—C11—C10	120.94 (18)	C2—C3—H3	120.4
C12—C11—H11	119.5	O1—C6—C1	118.05 (15)
C10—C11—H11	119.5	O1—C6—C5	121.05 (16)
C13—C14—C9	119.17 (17)	C1—C6—C5	120.90 (16)
C13—C14—H14	120.4	C6—O1—H1	109.5
C9—C14—H14	120.4	C1—C2—C3	122.26 (18)
C10—C9—C14	118.98 (17)	C1—C2—H2	118.9
C10—C9—N1	116.45 (16)	C3—C2—H2	118.9
C14—C9—N1	124.57 (16)	C2—C1—C6	117.95 (17)
C11—C10—C9	120.54 (18)	C2—C1—C7	121.44 (18)
C11—C10—H10	119.7	C6—C1—C7	120.61 (17)
C9—C10—H10	119.7	C1—C7—H7A	109.5
N1—C8—C5	122.65 (16)	C1—C7—H7B	109.5
N1—C8—H8	118.7	H7A—C7—H7B	109.5
C5—C8—H8	118.7	C1—C7—H7C	109.5
C8—N1—C9	123.07 (15)	H7A—C7—H7C	109.5
C3—C4—C5	121.12 (18)	H7B—C7—H7C	109.5
C3—C4—H4	119.4		
			
C11—C12—C13—C14	−0.3 (3)	N1—C8—C5—C4	176.22 (18)
C11—C12—C13—Cl1	−179.48 (15)	N1—C8—C5—C6	−2.7 (3)
C13—C12—C11—C10	0.6 (3)	C5—C4—C3—C2	0.3 (3)
C12—C13—C14—C9	−0.6 (3)	C4—C5—C6—O1	179.49 (16)
Cl1—C13—C14—C9	178.56 (14)	C8—C5—C6—O1	−1.6 (3)
C13—C14—C9—C10	1.2 (3)	C4—C5—C6—C1	−0.5 (3)
C13—C14—C9—N1	−178.54 (16)	C8—C5—C6—C1	178.43 (16)
C12—C11—C10—C9	−0.1 (3)	C4—C3—C2—C1	−0.5 (3)
C14—C9—C10—C11	−0.9 (3)	C3—C2—C1—C6	0.2 (3)
N1—C9—C10—C11	178.88 (16)	C3—C2—C1—C7	179.21 (19)
C5—C8—N1—C9	−179.81 (15)	O1—C6—C1—C2	−179.65 (16)
C10—C9—N1—C8	−176.76 (17)	C5—C6—C1—C2	0.3 (3)
C14—C9—N1—C8	3.0 (3)	O1—C6—C1—C7	1.3 (3)
C3—C4—C5—C6	0.2 (3)	C5—C6—C1—C7	−178.70 (17)
C3—C4—C5—C8	−178.79 (17)		

### Hydrogen-bond geometry (Å, º)

*Cg*1 and *Cg* are the centroids of the C1–C6 and C9–C14 rings, respectively.

**Table:**

*D*—H···*A*	*D*—H	H···*A*	*D*···*A*	*D*—H···*A*
O1—H1···N1	0.82	1.88	2.605 (2)	148
C10—H10···O1^i^	0.93	2.56	3.402 (2)	151
C2—H2···*Cg*1^ii^	0.93	2.75	3.561 (2)	147
C12—H12···*Cg*2^iii^	0.93	2.78	3.589 (2)	147

Symmetry codes: (i) −*x*+1, *y*+1/2, −*z*+1/2; (ii) −*x*+3/2, *y*−1/2, *z*; (iii) −*x*+1/2, *y*+1/2, *z*.

## References

[bb1] Dennington, R. I. I., Keith, T. & Millam, J. (2007). *GaussView.* Version 4.1.2. Semichem Inc., Shawnee Mission, KS, USA.

[bb2] Elmalı, A. & Elerman, Y. (1998). *J. Mol. Struct.* **442**, 31–37.

[bb3] Farrugia, L. J. (2012). *J. Appl. Cryst.* **45**, 849–854.

[bb4] Frisch, M. J., *et al.* (2009). *GAUSSIAN09*. Gaussian Inc., Wallingford, CT, USA.

[bb5] Fun, H.-K., Quah, C. K., Viveka, S., Madhukumar, D. J. & Nagaraja, G. K. (2011). *Acta Cryst.* E**67**, o1934. 10.1107/S1600536811026122 PMC3212321 22090978

[bb6] Groom, C. R., Bruno, I. J., Lightfoot, M. P. & Ward, S. C. (2016). *Acta Cryst.* B**72**, 171–179. 10.1107/S2052520616003954 PMC4822653 27048719

[bb7] Jafari, H., Danaee, I., Eskandari, H. & RashvandAvei, M. (2013). *Ind. Eng. Chem. Res.* **52**, 6617–6632. 10.1080/10934529.2013.815094 23947700

[bb8] Karakaş, A., Elmali, A., Ünver, H. & Svoboda, I. (2004). *J. Mol. Struct.* **702**, 103–110.

[bb9] Karunakaran, V. & Balachandran, V. (2014). *Spectrochim. Acta A Mol. Biomol. Spectrosc.* **128**, 1–14. 10.1016/j.saa.2014.02.155 24657464

[bb10] Koepnick, B. D., Lipscomb, J. S. & Taylor, D. K. (2010). *J. Phys. Chem. A*, **114**, 13228–13233. 10.1021/jp108619n 21090601

[bb11] Macrae, C. F., Edgington, P. R., McCabe, P., Pidcock, E., Shields, G. P., Taylor, R., Towler, M. & van de Streek, J. (2006). *J. Appl. Cryst.* **39**, 453–457.

[bb12] Özek, A., Albayrak, Ç., Odabaşoğlu, M. & Büyükgüngör, O. (2009). *J. Chem. Crystallogr.* **39**, 353–357.

[bb14] Politzer, P. & Murray, J. S. (2002). *Theor. Chimi. Acta*, **108**, 134–142.

[bb15] Prabavathi, N., Senthil, N. N. & Krishnakumar, V. (2015). *Pharm Anal Acta* **6**, 1–20.

[bb13] Przybylski, P., Huczynski, A., Pyta, K., Brzezinski, B. & Bartl, F. (2009). *Curr. Org. Chem.* **13**, 124–148.

[bb16] Schiff, H. (1864). *Ann. Chem. Suppl*, **3**, 343–349.

[bb17] Sheldrick, G. M. (2015*a*). *Acta Cryst.* A**71**, 3–8.

[bb18] Sheldrick, G. M. (2015*b*). *Acta Cryst.* C**71**, 3–8.

[bb19] Sinha, D., Tiwari, A. K., Singh, S., Shukla, G., Mishra, P., Chandra, H. & Mishra, A. K. (2008). *Eur. J. Med. Chem.* **43**, 160–165. 10.1016/j.ejmech.2007.03.022 17532543

[bb20] Solomon, R. V., Bella, A. P., Vedha, S. A. & Venuvanalingam, P. (2012). *Phys. Chem. Chem. Phys. PCCP*, **14**, 14229–14237. 10.1039/c2cp41554b 22847369

[bb21] Spek, A. L. (2009). *Acta Cryst.* D**65**, 148–155. 10.1107/S090744490804362X PMC2631630 19171970

[bb22] Stoe & Cie (2002). *X-AREA* and *X-RED32*. Stoe & Cie GmbH, Darmstadt, Germany.

[bb23] Tahir, M. N., Shad, H. A., Rauf, A. & Khan, A. H. (2015). *Acta Cryst.* E**71**, o137–o138. 10.1107/S2056989015001401 PMC4384559 25878871

[bb24] Yılmaz, I., Kazak, C., Gümüş, S., Ağar, E. & Ardalı, Y. (2012). *Spectrochim. Acta A Mol. Biomol. Spectrosc.* **97**, 423–428. 10.1016/j.saa.2012.06.032 22820045

[bb25] Zheng, Y. (2013*a*). *Acta Cryst.* E**69**, o1190. 10.1107/S1600536813017558 PMC3770443 24046728

[bb26] Zheng, Y.-F. (2013*b*). *Acta Cryst.* E**69**, o1349. 10.1107/S1600536813019454 PMC3793828 24109415

